# Editorial: The role of the bone marrow microenvironment in multiple myeloma evolution and therapy

**DOI:** 10.3389/fonc.2023.1157555

**Published:** 2023-02-21

**Authors:** Carolina Schinke, Niels Weinhold, Jesus Delgado-Calle

**Affiliations:** ^1^ Department of Hematology-Oncology Myeloma Center, University of Arkansas for Medical Sciences, Little Rock, AR, United States; ^2^ Department of Hematology-Oncology Systems Medicine & Computational Biology, Heidelberg University, Heidelberg, Germany; ^3^ Department of Physiology and Cell Biology, University of Arkansas for Medical Sciences, Little Rock, AR, United States

**Keywords:** multiple myeloma, bone marrow microenviroment, bone marrow niche, bone metabolism, mesenchymal stem cells, SPARC, dormant myeloma cells, sphingolipid metabolism

Multiple myeloma (MM) is a hematopoietic malignancy of terminally differentiated plasma cells that reside within the bone marrow (BM) and heavily rely on the surrounding BM microenvironment (BM-ME) for disease progression. MM cells locate in specialized niches in the BM where they interact with cells of the tumor microenvironment, transforming the BM-ME into an ideal niche for the migration, proliferation, and survival of MM cells. Although survival rates have improved in the past decade, almost all patients relapse, ultimately resulting in treatment resistance and death. MM evolution and treatment resistance arise due to intrinsic mechanisms as well as cellular interactions within the BM-ME that promote tumor growth and survival. Well known clinical examples of the contribution of the dysregulated BM-ME in MM patients to MM disease progression are the commonly observed formation of focal lytic lesions due to increased bone resorption, which persist even during disease remission, and the presence of immuno-paresis as an example of aberrant immune function. Targeting the BM-ME has hence become a vital treatment strategy in MM patients. Yet, due to the complex cellular and molecular mechanisms mediating the crosstalk between MM cells and the BM and the lack of comprehensive biological functions of specific BM components, our understanding remains limited and continuous research is urgently needed in this field. This special issue attempts to deepen our knowledge of interactions between MM and its BM-ME and guide the development of potential novel therapeutic targets to treat MM.

Interleukin 17A (IL-17A), a key cytokine in T-cell activation, is elevated in MM patients, and previous reports have indicated a potential therapeutic role of targeting IL-17A. In this issue, Dong et al. provide evidence that IL-17A might play an important role in the formation of MM bone disease. The authors show that increased levels of serum IL-17A are independently correlated with higher severity of bone disease and fracture incidence in newly diagnosed MM patients. Interestingly, high IL-17A serum levels were also associated with poor progression-free and overall survival, albeit only in patients with light chain disease and IgA MM. These results support the potential use of IL-17A targeted therapy in the future.

Mesenchymal stem cells (MSCs) represent an integral part of the BM-ME and promote the proliferation and survival of MM cells. Yet, therapeutic interventions to target MSCs are not clinically available. In their study, Heinemann et al. investigated the transcriptome of MSCs in patients with active MM compared to patients in complete remission (CR) or control subjects. The transcriptome of MSCs from patients with active MM was significantly enriched for genes associated with the PI3K-ACT-mTOR signaling pathway, which was also confirmed at the protein level. Treatment with the pan-PI3K inhibitor pictilisib selectively reduced the growth of MSCs from patients with active MM compared to those from CR or control subjects. Furthermore, pictilisib fully abrogated the proliferation supporting role of MSCs derived from active MM patients on the growth of MM cell lines, highlighting the therapeutic potential of targeting this pathway in the MM BM-ME.

Further, Aly et al. investigated the role of SPARC (secreted protein acidic and rich in cysteine) in myelomagenesis. SPARC is a common stromal motif expressed by follicular dendritic cells. It regulates numerous cellular processes, including immune cell networking and extracellular matrix assembly in lymphoid tissue and BM. The authors show that SPARC expression was significantly higher in primary BM samples compared to lymphoid tissues, and its expression correlated inversely with the level of plasma cell infiltration. *In-vitro*, co-culture of SPARC-expressing follicular dendritic cells with lymphocytes inhibited the expression of several oncogenes associated with malignant transformation to plasma cells, underscoring an important role of SPARC expression in myelomagenesis.

Additionally, this special issue includes two reviews on the BM-ME in MM. Dadzie and Green elegantly summarize the role of the BM niche in MM survival and evolution. Of particular emphasis is the importance of the BM-ME to allow MM cells to enter a quiescent or dormant state, contributing to treatment evasion and regrowth at a later stage. Petrusca et al. review recent advances in biological implications of sphingolipid metabolism alterations in MM evolution. Sphingolipids are complex bioactive lipids involved in nearly all cellular functions. Recent studies have emphasized that sphingolipid metabolism and function are significantly altered in the MM BM-ME, which can contribute to the formation of bone lesions and drug resistance. The manuscript also highlights how the aberrant sphingolipid metabolism can be used for prognostic and therapeutic strategies.

Taken together, the studies presented in this Research Topic highlight the importance and complexity of the supportive role of the BM-ME in the proliferation, drug resistance, and survival of MM cells. Advancing this research field will ultimately lead to improving prognostic factors and identifying new therapeutic options in MM. We thank all authors who have contributed to this interesting special issue.

## Author contributions

All authors listed have made a substantial, direct, and intellectual contribution to the work and approved it for publication.

